# Extracellular matrix components modulate different stages in β_2_-microglobulin amyloid formation

**DOI:** 10.1074/jbc.RA119.008300

**Published:** 2019-04-17

**Authors:** Núria Benseny-Cases, Theodoros K. Karamanos, Cody L. Hoop, Jean Baum, Sheena E. Radford

**Affiliations:** From the ‡ Astbury Centre for Structural Molecular Biology and School of Molecular and Cellular Biology, Faculty of Biological Sciences, University of Leeds, Leeds LS2 9JT, United Kingdom and; the § Department of Chemistry and Chemical Biology, Rutgers University, Piscataway, New Jersey 08854

**Keywords:** amyloid, protein aggregation, protein misfolding, collagen, extracellular matrix, fibril, β2-microglobulin, dialysis-related amyloidosis (DRA), glycosaminoglycan, heparin, MHC I

## Abstract

Amyloid deposition of WT human β_2_-microglobulin (WT-hβ_2_m) in the joints of long-term hemodialysis patients is the hallmark of dialysis-related amyloidosis. *In vitro*, WT-hβ_2_m does not form amyloid fibrils at physiological pH and temperature unless co-solvents or other reagents are added. Therefore, understanding how fibril formation is initiated and maintained in the joint space is important for elucidating WT-hβ_2_m aggregation and dialysis-related amyloidosis onset. Here, we investigated the roles of collagen I and the commonly administered anticoagulant, low-molecular-weight (LMW) heparin, in the initiation and subsequent aggregation phases of WT-hβ_2_m in physiologically relevant conditions. Using thioflavin T fluorescence to study the kinetics of amyloid formation, we analyzed how these two agents affect specific stages of WT-hβ_2_m assembly. Our results revealed that LMW-heparin strongly promotes WT-hβ_2_m fibrillogenesis during all stages of aggregation. However, collagen I affected WT-hβ_2_m amyloid formation in contrasting ways: decreasing the lag time of fibril formation in the presence of LMW-heparin and slowing the rate at higher concentrations. We found that in self-seeded reactions, interaction of collagen I with WT-hβ_2_m amyloid fibrils attenuates surface-mediated growth of WT-hβ_2_m fibrils, demonstrating a key role of secondary nucleation in WT-hβ_2_m amyloid formation. Interestingly, collagen I fibrils did not suppress surface-mediated assembly of WT-hβ_2_m monomers when cross-seeded with fibrils formed from the N-terminally truncated variant ΔN6-hβ_2_m. Together, these results provide detailed insights into how collagen I and LMW-heparin impact different stages in the aggregation of WT-hβ_2_m into amyloid, which lead to dramatic effects on the time course of assembly.

## Introduction

Dialysis-related amyloidosis (DRA)^5^ is a severe condition that leads to progressive bone and joint atrophy in the majority of long-term hemodialysis patients (1–5). This disorder results from the deposition of amyloid plaques formed predominantly of WT human β_2_-microglobulin (WT-hβ_2_m) in joints and cartilage tissue (3, 6–8). In its nonpathogenic role, WT-hβ_2_m constitutes the light chain of the major histocompatibility complex class I, which functions in presenting antigens to T-cells (9). After dissociation from the major histocompatibility complex class I complex, WT-hβ_2_m is normally degraded and excreted by the kidneys (10). In renal failure, the concentration of β_2_m in the plasma is increased up to >60 times compared with that of healthy individuals (3, 6–8). Aggregation of WT-hβ_2_m then leads to the formation of amyloid plaques that are deposited almost exclusively in skeletal tissues (11), which are rich in extracellular matrix (ECM) components, including collagens and the glycosaminoglycans (GAGs) heparan sulfate and hyaluronic acid (12–15). The mechanism of recruitment of WT-hβ_2_m specifically to skeletal tissues is not fully understood, but ECM components, such as low molecular weight (LMW)-heparin (a GAG mimic, relevant here because this is given to all patients undergoing renal replacement therapy), apolipoprotein E, and collagen, have been found to enhance WT-hβ_2_m aggregation *in vitro* (13, 16–18). Amyloid fibrils have also been found associated with collagen fibrils in *ex vivo* deposits from DRA patients (13), and monomers of both WT-hβ_2_m and its natural proteolytic product, ΔN6-hβ_2_m, which lacks the N-terminal six amino acids, have been shown to have weak (*K_D_*: 4.1 × 10^−4^ m and 4.9 × 10^−6^ m, respectively (15)) affinities for collagen I at the pathophysiologic pH of 6.4 (12). Despite this evidence of the importance of LMW-heparin and collagen in amyloid formation, the mechanism(s) by which interactions with the reagents affect aggregation of WT-hβ_2_m and ΔN6-hβ_2_m remain unclear.

Compared with the intransigence of WT-hβ_2_m to form amyloid fibrils at pH 6–7 (19, 20), ΔN6-hβ_2_m readily forms amyloid at these pH values *in vitro* (21, 22). ΔN6-hβ_2_m comprises ∼30% of the hβ_2_m present in DRA deposits (21, 23) and contains a nonnative *trans*-X-P32, a prerequisite for amyloid formation (22, 24) that is retained in the amyloid fibril structure itself (25). Weak interactions between the apical loops of ΔN6-hβ_2_m and WT-hβ_2_m have been shown to promote amyloid formation of the normally innocuous WT-hβ_2_m (26, 27), suggesting a potential role of ΔN6-hβ_2_m in initiating fibril assembly of the WT protein. How ΔN6-hβ_2_m, collagen, and LMW-heparin together influence amyloid formation of WT-hβ_2_m, however, has remained unclear.

Here we used detailed analysis of the kinetics of amyloid formation to determine the role of collagen I, LMW-heparin, and ΔN6-hβ_2_m and their mixtures on amyloid fibril formation of WT-hβ_2_m. The results reveal that LMW-heparin and collagen I influence multiple phases of WT-hβ_2_m amyloid formation, including initiation, elongation, and secondary nucleation processes. Additionally, we found that the effects of collagen I on amyloid formation depend on whether fibril growth of WT-hβ_2_m is self-seeded or cross-seeded by ΔN6-hβ_2_m fibrils. Overall, the results shed new light on the mechanisms by which biologically relevant factors influence WT-hβ_2_m amyloid assembly. More generally, they reveal how the local environment can have a dramatic effect in defining the rate and mechanisms of protein assembly into amyloid.

## Results

### LMW-heparin and collagen I have a synergistic effect in the initiation of amyloid formation of WT-hβ_2_m

**Figure 1.**
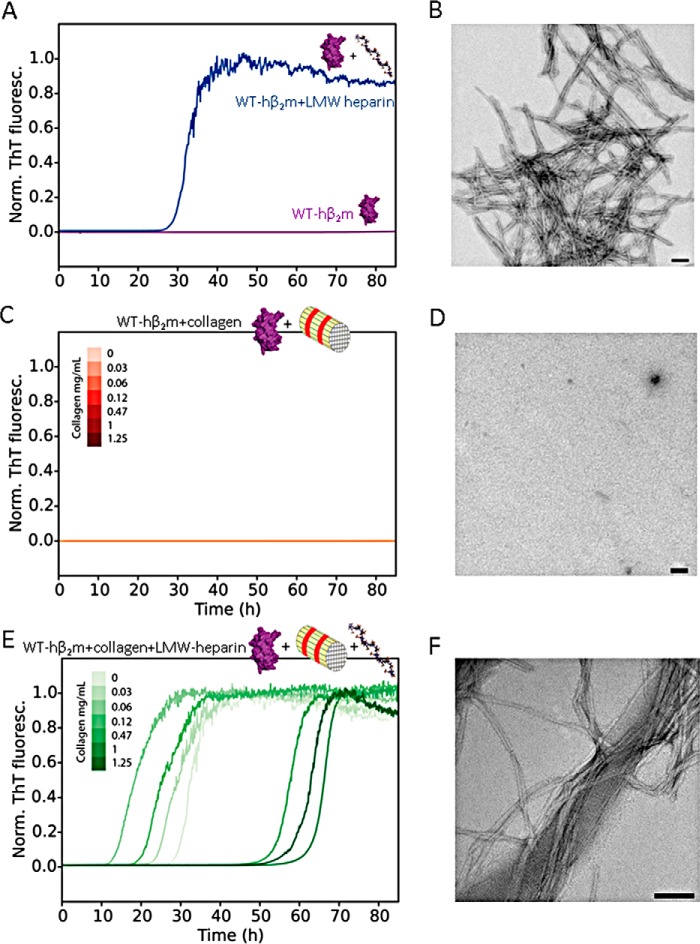
*A*, amyloid fibril formation of WT-hβ_2_m (40 μm) in the absence (*purple*) or presence (*blue*) of 0. 1 mg/ml LMW-heparin. *B*, negative stain TEM at the end of the aggregation reaction (80 h) for WT-hβ_2_m in the presence of 0.1 mg/ml LMW-heparin. *C*, effect of collagen I on WT-hβ_2_m aggregation in the absence of LMW-heparin. Collagen I concentrations are indicated in the *panel* in mg/ml. *D*, negative stain TEM of the sample at 1 mg/ml collagen after 80 h of incubation. *E*, effect of collagen I on WT-hβ_2_m aggregation in the presence of 0.1 mg/ml LMW-heparin. For each condition, a single ThT fluorescence (*Norm. ThT fluoresc.*) trace representative of the mean aggregation kinetics taken over at least three replicate experiments with three samples in each is shown (see also Fig. S1). *F*, representative negative stain TEM of the end point of the aggregation process in the presence of 1 mg/ml collagen I and 0.1 mg/ml LMW-heparin. *Scale bar* in all TEM panels indicates 100 nm. Note the schematic drawings used to annotate the different reagents present in different experiments (WT-hβ_2_m (*purple*), LMW-heparin (*gray*), and collagen I fibrils (*yellow/red bundle*)). These *symbols* are used throughout the manuscript to denote the additives included in each experiment.

The hallmark of DRA is formation of proteinaceous deposits comprised of WT-hβ_2_m and ΔN6-hβ_2_m in the ECM-rich joint spaces (3). Because native WT-hβ_2_m does not form amyloid at neutral pH or at the slightly acidified pH (pH 6.2) in affected joints (18) unless co-solvents or copper ions are added (13, 16–19, 28–32) (Fig. 1*A*), we investigated how collagen I that is found in the ECM and GAGs (represented by LMW-heparin) affect the kinetics of aggregation of WT-hβ_2_m. Previous studies have demonstrated a role of these components in hβ_2_m amyloid assembly (13, 15, 17, 18, 33), but the precise mechanism(s) by which they affect aggregation, and the possible synergy between these different components, remained unclear. Using the enhancement of thioflavin T (ThT) fluorescence as a probe of amyloid formation, measurement of the resulting fibril growth kinetics showed that LMW-heparin (0.1 mg/ml) induces fibril formation of WT-hβ_2_m (0.47 mg/ml) within ∼30 h, resulting in the formation of long, straight fibrils typical of amyloid (Fig. 1, *A* and *B*). By contrast, collagen I did not induce amyloid formation in the absence of LMW-heparin over the time scale measured here (Fig. 1, *C* and *D*) (note, however, that collagen can promote amyloid fibril formation over much longer time scales, as previously reported (13)). The addition of both components revealed that collagen I modulates the kinetics of LMW-heparin–driven WT-hβ_2_m fibril formation in a complex manner (Fig. 1, *E* and *F*). At low concentrations (0.03–0.12 mg/ml), collagen I accelerates LMW-heparin–induced aggregation of WT-hβ_2_m, decreasing the lag time relative to the effect of LMW-heparin alone (Fig. 1*E*, *light green colors*, compared with Fig. 1*A*, *blue*). However, the addition of higher concentrations of collagen I (≥0.47 mg/ml) in the presence of LMW-heparin retards fibril formation by increasing the lag time (Fig. 1*E*, *dark green colors*; see also Fig. S1*A*). Enhancement of WT-hβ_2_m amyloid formation by collagen I is consistent with previous results, which have shown that collagen I alone can induce aggregation of WT-hβ_2_m (13). WT-hβ_2_m has also been shown to bind to both collagen I and LMW-heparin (12, 14, 15, 18). At high concentrations of collagen I, we assume that competition between collagen I and LMW-heparin binding to each other (*K_D_*: 7.9 × 10^−8^ m) (34), and binding to WT-hβ_2_m may reduce the availability of these components to interact with WT-hβ_2_m slowing its aggregation. Sequestration of WT-hβ_2_m nuclei/aggregates on the surface of the collagen I fibrils, which would be favored at high concentrations of collagen I, could also disfavor amyloid formation (see below) and contribute to the complex dose-dependent behavior observed.

### Role of the collagen sequence and conformation in LMW-heparin–induced aggregation of WT-hβ_2_m

**Figure 2.**
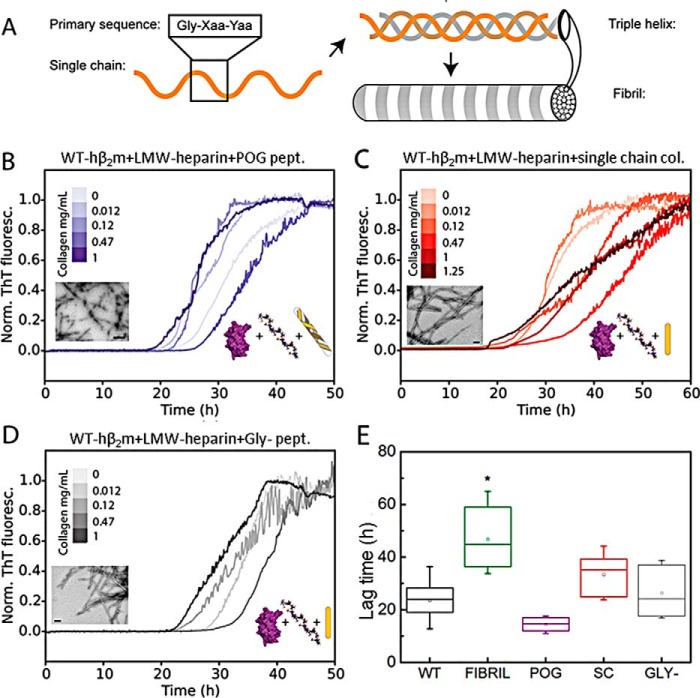
**The effect of CMPs and denatured collagen chains on LMW-heparin–induced WT-hβ_2_m fibril formation.** *A*, schematic of collagen hierarchical conformations, from the primary amino acid sequence, single-chain polyproline type II helix, triple helix of three single chains, and higher order fibrils. *B–D*, LMW-heparin–induced aggregation kinetics of WT-hβ_2_m (40 μm) in the presence of POG_10_ peptide (which forms a stable collagen triple helix; *B*), collagen I in the single chain form (*C*), and Gly− peptide (which does not form a stable triple helix; *D*). The concentration of the peptides added is indicated by *color* in the *inset*. All *traces* were obtained with 0.1 mg/ml LMW-heparin. Negative stain TEM micrographs of the end points are shown as an *inset* for each condition. *Scale bar*, 100 nm. *E*, box plot of the lag time for the WT-hβ_2_m aggregation in the presence or absence of 1 mg/ml collagen I fibrils (data taken from Fig. 1*E*), denatured collagen, or CMPs. *WT*, WT-hβ_2_m alone (no collagen or CMPs added); *FIBRIL*, WT-hβ_2_m plus collagen I fibrils; *POG*, WT-hβ_2_m plus POG_10_ peptide; *SC*, WT-hβ_2_m plus single chain collagen I; *GLY*−, WT-hβ_2_m plus Gly− peptide. The data are representative of three replicate experiments, with three samples in each. *Asterisk* denotes *p* < 0.002. *Norm. ThT fluoresc.*, normal ThT fluorescence.

Collagen I can adopt a hierarchy of structures within the ECM (Fig. 2*A*). The canonical collagenous sequence consists of Gly-Xaa-Yaa triplets, where Xaa and Yaa can be any amino acid but are most often Pro and hydroxyproline (Hyp/O), respectively. Single collagen polypeptide chains fold into polyproline type II helices. Three such chains then twist together to form a triple helix that is stabilized by a network of interchain hydrogen bonds between Gly and Xaa of a neighboring chain (35). The triple helices further self-assemble into higher order fibrils. To determine the effects of collagen sequence and conformation on the aggregation kinetics of WT-hβ_2_m, the kinetics of LMW-heparin–induced aggregation were monitored in the presence of two different collagen mimetic peptides (CMPs), as well as denatured full-length collagen I. The CMP, Ac-(Pro-Hyp-Gly)_10_-GY-NH_2_ (named POG_10_), forms a stable triple helix conformation without additional native sequence fragments (36). However, this peptide does not form intermolecular interactions required to proceed to collagen fibril formation. Removal of only one Gly from the middle POG repeat (named Gly−) disrupts the triple helix conformation (37, 38). By contrast with the complex effects of collagen I fibrils on the lag time of LMW-heparin–induced aggregation of WT-hβ_2_m (Fig. 1*E*), incubation of WT-hβ_2_m monomers (0.47 mg/ml) with up to 1 mg/ml POG_10_ or Gly− peptide, in the presence of 0.1 mg/ml LMW-heparin did not significantly affect the lag time of fibril formation relative to the lag time in the absence of peptide (Fig. 2, *B*, *D*, and *E*, and Fig. S1, *A*, *B*, and *D*). Full-length collagen I denatured into single chains also had no significant effect on the lag time of aggregation (Fig. 2, *C* and *E*, and Fig. S1*C*). Hence, adoption of a triple helical collagen structure with a native sequence is required for collagen I to modulate the rate of LMW-heparin–induced WT-hβ_2_m aggregation, highlighting the specificity between the different surfaces involved in amyloid assembly.

### Surface-mediated aggregation of WT-hβ_2_m is protected by collagen I

**Figure 3.**
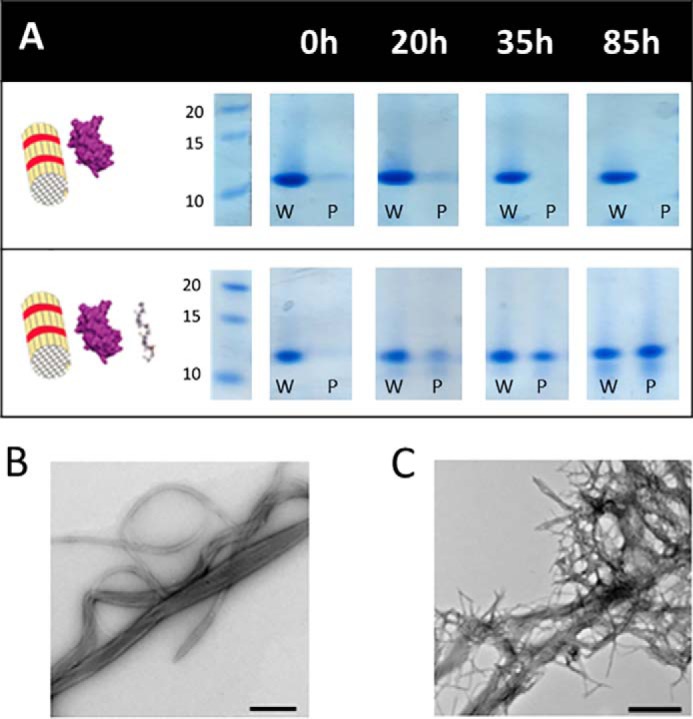
*A*, co-pelleting of WT-hβ_2_m aggregated for different times with collagen I fibrils with/without 0.1 mg/ml LMW-heparin. SDS-PAGE analysis of the whole sample (*w*) and the pellet after sedimentation of collagen I by low-speed centrifugation (*P*). Molecular mass markers with masses in kDa are shown alongside. *B*, negative stain TEM micrograph of WT-hβ_2_m incubated in the presence of 1.0 mg/ml collagen I and 0.1 mg/ml LMW-heparin for 15 h, showing the presence of collagen I fibrils but no WT-hβ_2_m amyloid. *C*, as in *B*, but image taken after incubation for 65 h. WT-hβ_2_m amyloid can be clearly seen in this image alongside collagen I fibrils. The *scale bar* in each micrograph is 500 nm.

To determine whether a stable complex is formed between WT-hβ_2_m monomer and/or fibrils and collagen I, samples were taken at different times during aggregation in the presence or absence of 0.1 mg/ml LMW-heparin, added to collagen I (1.0 mg/ml), and pelleted 10 min later by centrifugation at 5000 × *g* (see “Experimental procedures”). At this low centrifugation speed, only collagen I fibrils sediment, whereas WT-hβ_2_m (monomers, oligomers, and fibrils) remain in the supernatant. Whether WT-hβ_2_m monomers/small oligomers and/or fibrils bind collagen I was then determined by monitoring the band intensity of WT-hβ_2_m that pelleted with collagen I on an SDS-PAGE gel. These experiments showed that WT-hβ_2_m co-precipitates with collagen I fibrils only after an incubation time of 35 h (Fig. 3, *A–C*) (approximately the t_50_ of aggregation in the presence of 0.1 mg/ml LMW-heparin (Fig. 1*A*)). A higher extent of co-precipitation was observed after 85 h, by which time WT-hβ_2_m amyloid formation has reached completion in the presence of LMW-heparin (Figs. 1*A* and 3, *A* and *C*). These results show that the fibrillar form of WT-hβ_2_m interacts with collagen I fibrils most tightly, whereas species formed in the lag time appear not to bind collagen I tightly, at least at the detection limit of these experiments. Consistent with these results, interactions between WT-hβ_2_m monomers and LMW-heparin or collagen I were found to be weak as assessed by ^1^H-^15^N heteronuclear single quantum coherence NMR spectra. This was assessed by 1 h co-incubation of 80 μm ^15^N-WT-hβ_2_m with 0.2 mg/ml LMW-heparin and/or 2 mg/ml collagen I and measurement of the chemical shifts of backbone resonances. No significant chemical shift perturbations (or linewidth) were observed in these experiments (Fig. S2), suggesting that these interactions are too weak to detect by these methods under the conditions employed.

**Figure 4.**
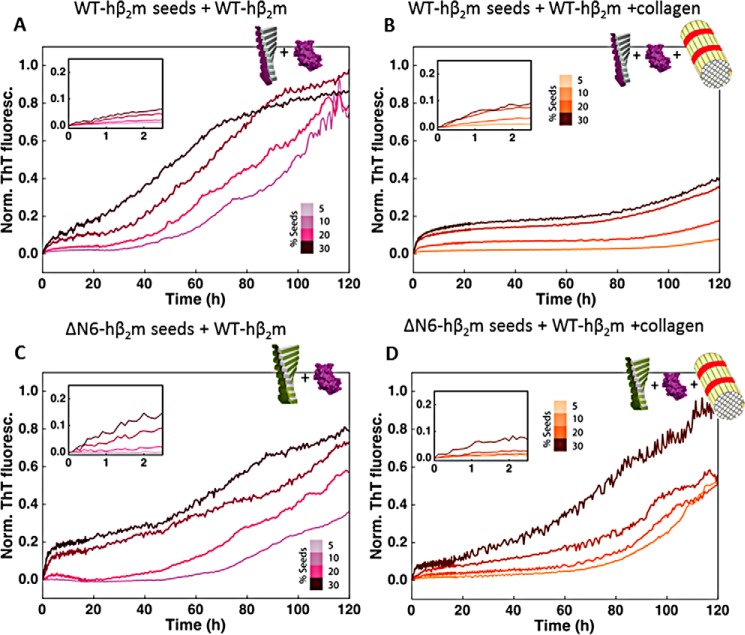
**The effect of collagen I fibrils on self-seeded and cross-seeded growth with WT-hβ_2_m monomers.** *A*, aggregation kinetics of WT-hβ_2_m in the presence of preformed WT-hβ_2_m seeds (5–30%, v/v). The *inset* shows an expanded plot over the first ∼2 h. *B*, as in *A*, but in the presence of 1 mg/ml collagen I. *C*, aggregation kinetics of WT-hβ_2_m in the presence of preformed ΔN6-hβ_2_m seeds (5–30%, v/v). The *inset* shows an expanded plot over the first ∼2 h. *D*, as in *C*, but upon addition of 1 mg/ml of collagen I. Three replicate experiments, with three samples in each were measured. Here, a single trace representative of the mean aggregation kinetics is shown. See also Fig. S3 for statistics. *Norm. ThT fluoresc.*, normal ThT fluorescence.

Because WT-hβ_2_m fibrils and collagen I were shown to interact by the co-pelleting assay, we next analyzed the effect of collagen I on seeded growth of WT-hβ_2_m fibrils (using WT-hβ_2_m fibril seeds produced in the presence of LMW-heparin (see “Experimental procedures”)). As expected, the addition of WT-hβ_2_m seeds (5–30%, v/v) enhances the rate of formation of WT-hβ_2_m fibrils, dependent on the seed concentration (Fig. 4*A*). Note that under these quiescent conditions and without seeds, no fibrils form (22, 24, 26). Interestingly, a biphasic curve is generated in the presence of fibril seeds, with the first (relatively small) increase in ThT fluorescence intensity occurring in the first 2 h (Fig. 4, *A* and *inset*). This phase presumably monitors the elongation of fibril ends by WT-hβ_2_m monomers. The addition of 1 mg/ml collagen I does not affect this phase (Fig. 4*B*). By contrast, the second phase, with larger ThT amplitude (Fig. 4*A*), is significantly retarded by the addition of 1 mg/ml collagen I (Fig. 4*B* and Fig. S3). In the presence of low concentrations (5%, v/v) of seeds and under quiescent conditions, these fibril-mediated interactions are the dominant processes of fibril formation. Hence, in these conditions, the interaction of collagen I with WT-hβ_2_m fibrils, observed by co-precipitation (Fig. 3*A*), protects against fibril surface-mediated growth of WT-hβ_2_m amyloid by masking the fibril surface.

The specificity of collagen I for WT-hβ_2_m fibrils was next probed by monitoring the effect of collagen I on reactions in which preformed seeds of ΔN6-hβ_2_m were used to cross-seed amyloid formation of WT-hβ_2_m monomers (Fig. 4, *C* and *D*). WT-hβ_2_m and ΔΝ6-hβ_2_m are known to co-aggregate (13, 18, 23), forming fibrils of a different morphology *in vitro* than those formed by each protein alone (27). The results showed that the effect of collagen I on the seeded aggregation of WT-hβ_2_m is highly dependent on the identity of the seeds added. The addition of ΔΝ6-hβ_2_m seeds to WT-hβ_2_m monomers also results in biphasic fibril growth curves (Fig. 4*C*). However, the rate of the initial phase is slower under all conditions for the cross-seeded reactions compared with the self-seeded reactions (Fig. S3*A*). Most notably, the secondary process, which occurs after rapid fibril elongation, is much less affected by collagen I in the cross-seeded reactions than when self-seeded (Fig. 4, compare *B* and *D*; see also Fig. S3*B*). Thus, the interference of collagen I with surface-mediated growth of WT-hβ_2_m fibrils depends on the morphology of the hβ_2_m fibril seeds, which differ when self-seeded and cross-seeded by ΔΝ6-hβ_2_m (27).

### LMW-heparin promotes assembly of WT-hβ_2_m fibrils in both self-seeded and cross-seeded reactions

**Figure 5.**
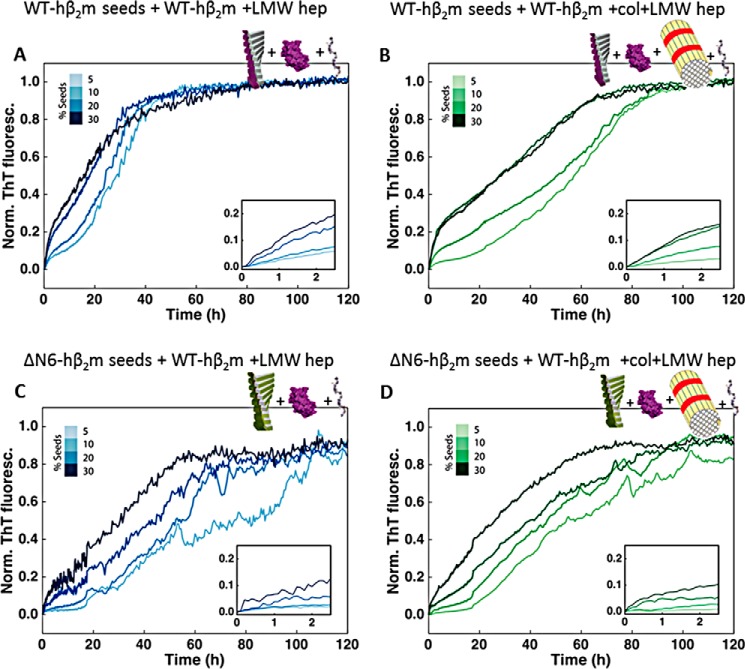
**The effect of LMW-heparin on self-seeded and cross-seeded growth with WT-hβ_2_m monomers.** *A*, aggregation kinetics of WT-hβ_2_m in the presence of preformed WT-hβ_2_m seeds (5–30%, v/v) and 0.1 mg/ml LMW-heparin. The *inset* shows an expansion of the first ∼2 h. *B*, as in *A*, but in the presence of 0.1 mg/ml LMW-heparin and 1 mg/ml collagen I. *C*, aggregation kinetics of WT-hβ_2_m in the presence of preformed ΔN6-hβ_2_m seeds (5–30%, v/v) in the presence of 0.1 mg/ml LMW-heparin. The *inset* shows an expansion of the first ∼2 h. *D*, as in *C*, but in the presence of 0.1 mg/ml LMW-heparin and 1 mg/ml collagen I. Three replicate experiments, with three samples in each were acquired. See also Fig. S3. *Norm. ThT fluoresc.*, normal ThT fluorescence.

Finally, the effect of LMW-heparin on fibril formation of WT-hβ_2_m was monitored in seeded reactions to determine whether the addition of this GAG can outcompete the effect of collagen I on aggregation. When mixed, LMW-heparin is able to rescue the inhibitory effect of collagen I on secondary processes whether the reaction is self- or cross-seeded (Fig. 5, *A–D*, and Fig. S3). Thus, collagen I and LMW-heparin have different effects on WT-hβ_2_m aggregation at multiple phases of fibrillation. Collagen I acts primarily on the secondary surface-mediated growth of self-seeded fibrils and depends on whether the reaction is self-seeded or cross-seeded (compare Fig. 4, *A* and *B*, with Fig. 5, *A* and *B*). By contrast, LMW-heparin can enhance growth at all stages of aggregation and is insensitive to the distinct amyloid conformations produced by self-seeding or cross-seeding with ΔN6-hβ_2_m.

## Discussion

A detailed study of the effects of the local environment on the course of protein aggregation is important for understanding how amyloid formation may be initiated and substantiated *in vivo*. Previous studies have shown the effects of chaperones (39–41), metal ions (19, 32, 42), membranes (43), trifluoroethanol or SDS co-solvents (28, 29, 44), GAGs (18, 30, 45, 46), and other natural compounds (13, 17, 47) on the formation of amyloid fibrils *in vitro*. For some of these compounds, detailed kinetic analysis has revealed the effect of each reagent on the different kinetic steps in aggregation, including primary nucleation, elongation from fibril ends, and secondary processes such as fibril fragmentation and secondary nucleation (39–41, 43, 48). Such studies can provide important information on the role of biologically relevant and other compounds on amyloid formation, including how the different factors may act synergistically to alter the course of aggregation in an *in vitro* setting (17, 30).

Here we have adopted this approach by examining the effects of two macromolecules that are present in conditions relevant to DRA, collagen I, and the GAG LMW-heparin. Because WT-hβ_2_m is not prone to amyloid formation in the absence of co-factors at pathophysiological pH and temperature, understanding how the molecular components of the local environment affect WT-hβ_2_m amyloid formation and impact the kinetics of fibril formation is important for developing an understanding of hβ_2_m amyloidogenesis in DRA. In addition, how the biological environment affects the ability of ΔN6-hβ_2_m, which makes up ∼30% of the hβ_2_m component in DRA plaques (21, 23), to stimulate aggregation of WT-hβ_2_m may also shed light on how the aggregation of WT-hβ_2_m may be initiated *in vivo*.

Understanding amyloid formation of WT-hβ_2_m in mechanistic detail in the context of the ECM in joints and cartilage is extremely challenging, given the multicomponent composition it presents. Here, we have started to investigate aggregation in this environment by determining how different components relevant to DRA (collagen I and LMW-heparin) impact the different kinetic stages of WT-hβ_2_m aggregation. The studies presented show that LMW-heparin, collagen I, and ΔN6-hβ_2_m have different effects on the course of WT-hβ_2_m aggregation, which compete for the different stages of aggregate formation. First, under all conditions, LMW-heparin is able to promote the self-assembly of WT-hβ_2_m, decreasing the lag time and increasing the rate of fibril formation by affecting secondary stages, whether self-seeded or cross-seeded by ΔN6-hβ_2_m. Previous studies have shown that LMW-heparin binds and stabilizes WT-hβ_2_m amyloid fibrils, whereas the nonsulfated GAG hyaluronic acid has no effect on fibril stability or the rate of fibril formation, suggesting that ionic interactions between the GAG and WT-hβ_2_m must be involved (17). Determining the origins of molecular recognition between different species (monomers, oligomers, and fibrils) of WT-hβ_2_m and ΔN6-β_2_m and GAGs will require further exploration, for example by varying the patterns of sulfation, the identity of the carbohydrate moieties that differ between GAGs, and the length of the GAG, which have been shown previously to affect amyloid-GAG recognition (46, 50). Analysis of the effects of heparan sulfate, the most abundant GAG in the joint ECM, would be particularly important for hβ_2_m, although previous studies have shown that heparan sulfate and LMW-heparin have similar effects on seeded elongation of fibril growth using WT-hβ_2_m (17).

**Figure 6.**
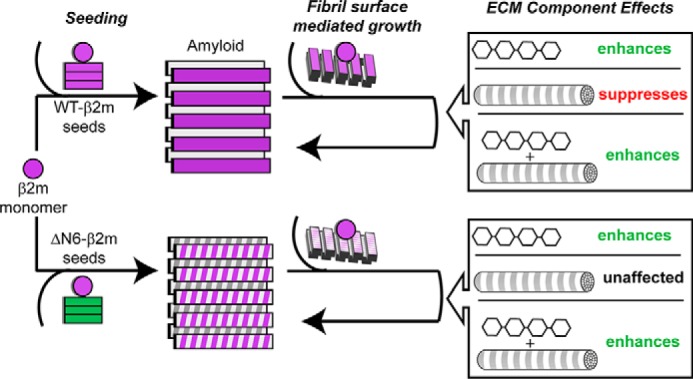
**Schematic of the effect of ECM components on seeded WT-hβ_2_m aggregation.** *Top row*, WT-hβ_2_m forms amyloid through self-seeded fibril growth. A secondary phase, fibril surface-mediated growth is enhanced by LMW-heparin and a co-mixture of LMW-heparin and collagen I but is suppressed by collagen alone. *Bottom row*, cross-seeding WT-hβ_2_m monomer with ΔN6-hβ_2_m seeds forms fibrils with a different conformation(27). The fibril surface mediated growth of these fibrils is also enhanced by LMW-heparin and the LMW-heparin and collagen I co-mixture but is not affected by collagen I alone.

By contrast with LMW-heparin, collagen I has a more complex role on WT-hβ_2_m assembly into amyloid, affecting the lag time of fibril formation and secondary growth phases in different ways, dependent on the concentration added, the presence of LMW-heparin, the structural organization of the collagen added, and whether fibril growth of WT-hβ_2_m is self-seeded or cross-seeded by ΔN6-hβ_2_m fibrils. A decrease in the lag time of WT-hβ_2_m assembly occurs upon addition of low concentrations of collagen I and LMW-heparin relative to the addition of LMW-heparin alone, suggestive of one route for the initiation of WT-hβ_2_m assembly at pathophysiological pH and temperature. However, at high concentrations of collagen I, the lag time is extended. Collagen I fibrils also interact strongly with WT-hβ_2_m amyloid fibrils, suppressing surface-mediated growth (Fig. 6, *top row*) by competing for interactions with the WT-hβ_2_m fibril surface. Notably, a different fibril morphology formed by cross-seeding WT-hβ_2_m with ΔN6-hβ_2_m fibrils does not show this marked suppression of fibril formation by collagen I (Fig. 6, *bottom row*). Whether self-seeded or cross-seeded with ΔN6 fibril seeds, LMW-heparin is able to outcompete the binding of collagen I to WT-hβ_2_m fibrils, releasing the potential of the amyloid fibril surface to enhance fibril formation via secondary nucleation processes. These results are consistent with previous studies that also showed enhanced aggregation of WT-hβ_2_m in the presence of LMW-heparin (18, 24). In addition to its role in suppressing amyloid formation, interactions between collagen I and WT-hβ_2_m fibrils may prevent the clearance of amyloid from the joint space, providing an explanation for the localization of DRA plaques to joint and cartilage tissues. Other factors not investigated here, such as the presence of chaperones and/or other proteins, oxidation, glycation, or other post-translational modifications of the hβ_2_m sequence, the presence of Cu^2+^ ions, and shear flow within the joint space, may also contribute to amyloid formation (17, 19, 31, 40, 51, 52). Overall, therefore, the results portray a marked complexity in amyloid formation in the ECM, in which a finely tuned balance of different components (in this case collagen I, LMW-heparin, and ΔN6-hβ_2_m) affect the progression of hβ_2_m aggregation and its sequestration in the joints to give a pattern of amyloid deposition that is the hallmark of DRA.

## Experimental procedures

### Protein preparation

WT-hβ_2_m and ΔN6-hβ_2_m were expressed and purified as described previously (22). For NMR experiments, ^15^N- and ^13^C-labeled WT-hβ_2_m and ΔN6 hβ_2_m were prepared as described in Ref. 53.

### Collagen preparation

Collagen type I (354249) from rat tail was purchased from BD Biosciences. Collagen was diluted to 3 mg/ml in 0.1 m acetic acid. Before use, it was dialyzed into 50 mm MES, 120 mm NaCl at pH 6.2. For preparation of collagen I fibrils, the dialyzed collagen was incubated at 37 °C for 1 h. For preparation of collagen single chains, the dialyzed collagen was incubated for 30 min at 70 °C. CMPs POG_10_ and Gly− were purchased from LifeTein and Tufts University Core facility, respectively, and were directly diluted into 50 mm MES, 120 mm NaCl at pH 6.2 to the concentrations specified.

### Formation of fibril seeds

Fibrils of WT-hβ_2_m were assembled in 50 mm MES buffer, 120 mm NaCl at pH 6.2 in the presence of 0.1 mg/ml LMW-heparin (Iduron) in a BMG Fluostar Optima plate reader at 37 °C at 600 rpm. The fibrils were sonicated for 1 min, distributed in aliquots, and frozen in liquid nitrogen. The size of the seeds was determined using negative stain transmission EM (TEM).

### Kinetic measurement of aggregation

WT-hβ_2_m and ΔN6-hβ_2_m fibrils were assembled in 50 mm MES buffer, 120 mm NaCl at pH 6.2. Fibril growth was performed in a BMG Fluostar Optima plate reader at 37 °C at 600 rpm (or quiescently in the case of seeded reactions). A final concentration of 10 μm ThT (Sigma) and 40 μm β_2_m was used. When required, seeds (5–30%, v/v) were added. The fibril yield was measured by centrifuging 50 μl of the end points at 14,000 × *g*, where amyloid fibrils are found in the pellet and soluble material remains in the supernatant and can be quantified spectrophotometrically.

### Determination of the lag time of fibril growth

The lag times of fibril growth under different conditions were determined by fitting a tangent to the curve at the midpoint of the elongation phase and extrapolating this time to the baseline signal in the lag phase. The intersection point of these two lines was considered the lag time.

### Determination of the elongation rate and the half-time of kinetics in the presence of seeds

ThT fluorescence curves were normalized to the final time point where ∼100% of the protein was converted to fibrils. In cases where this was not the case, and therefore there was no plateau in the fluorescence curves (such as in Fig. 4, *B* and *D*), the ThT signal was normalized to the corresponding value in the presence of LMW-heparin (Fig. 5, *B* and *D*) where the fibril yield was 100%. The observed elongation rate was calculated by fitting straight lines to the ThT fluorescence curves in the first hour of the normalized aggregation kinetics divided by the concentration of seeds. The *t*_50_ is the time taken to reach 50% of the maximal ThT fluorescence.

### Collagen co-precipitation

40 μm WT-hβ_2_m (in the presence/absence of 0.1 mg/ml LMW-heparin) was incubated at 37 °C at 600 rpm in a BMG Fluostar Optima plate reader. At different time points, 40-μl aliquots were taken and mixed with 1 mg/ml of collagen I fibrils and incubated for 10 min. The sample was centrifuged at 5000 × *g* for 10 min. Samples in the absence of LMW-heparin were used as a control. The pellet was washed once with the incubation buffer, and the centrifugation step was repeated. The pellet and the supernatant were then separately analyzed using 15% (w/v) polyacrylamide Tris-Tricine gels. The gels were stained with Coomassie Instant Blue (Expedeon).

### NMR spectroscopy

Samples of ^15^N-labeled protein (40–80 μm) in 50 mm MES buffer containing 120 mm NaCl, pH 6.2, 90% (v/v) H_2_O, 10% (v/v) D_2_O were used for NMR experiments. ^1^H-^15^N heteronuclear single quantum coherence spectra were collected in a Varian INOVA NMR spectrometer performing at 600 MHz and were processed in NMRPipe and analyzed using programs available in CCPNMR analysis (49, 54).

### EM

At the end of fibril assembly, 10 μl of sample were applied to carbon-coated EM grids. The grids were then carefully dried with filter paper before samples were negatively stained by the addition of 10 μl of 2% (w/v) uranyl acetate. Micrographs were recorded on a JEOL JEM-1400 electron microscope.

## Author contributions

N. B.-C., T. K. K., C. L. H., J. B., and S. E. R. conceptualization; N. B.-C. data curation; N. B.-C., C. L. H., J. B., and S. E. R. supervision; N. B.-C., C. L. H., J. B., and S. E. R. funding acquisition; N. B.-C., T. K. K., C. L. H., J. B., and S. E. R. investigation; N. B.-C., T. K. K., C. L. H., J. B., and S. E. R. methodology; N. B.-C., T. K. K., C. L. H., J. B., and S. E. R. writing-original draft; N. B.-C., C. L. H., J. B., and S. E. R. project administration; N. B.-C., T. K. K., C. L. H., J. B., and S. E. R. writing-review and editing; T. K. K., C. L. H., J. B., and S. E. R. formal analysis; T. K. K., C. L. H., J. B., and S. E. R. validation; T. K. K., C. L. H., J. B., and S. E. R. visualization.

## Supplementary Material

Supporting Information

## Footnotes

5 The abbreviations used are:

DRA: dialysis-related amyloidosis
hβ_2_m: human β_2_-microglobulin
ECM: extracellular matrix
ThT: thioflavin T
GAG: glycosaminoglycan
LMW: low molecular weight
CMP: collagen mimetic peptide
TEM: transmission electron microscopy
Tricine: *N*-[2-hydroxy-1,1-bis(hydroxymethyl)ethyl]glycine.
