# Supplementary material for: Extracellular matrix components modulate different stages in β2-microglobulin amyloid formation
Source: J Biol Chem. 2019 Apr 17;294(24):9392–401. doi: 10.1074/jbc.RA119.008300 (PMC6579475; doi:10.1074/jbc.RA119.008300)
Supplement: Supporting Information [file supp_294_24_9392__index.html]

Extracellular matrix components modulate different stages in β2-microglobulin amyloid formation — Extracellular matrix modulates β2m amyloid formation — Supporting Information 

# Extracellular matrix components modulate different stages in β2-microglobulin amyloid formation

## Supporting Information

- Supporting Information (to be published online) - Supporting Information
