# Supplementary material for: Extracellular matrix components modulate different stages in β2-microglobulin amyloid formation
Source: J Biol Chem. 2019 Apr 17;294(24):9392–401. doi: 10.1074/jbc.RA119.008300 (PMC6579475; doi:10.1074/jbc.RA119.008300)
Supplement: Supporting Information [file supp_RA119.008300_144124_2_supp_312850_pppkvx.pdf]

SUPPPORTING INFORMATION

Extracellular matrix components modulate different stages in β_2-microglobulin amyloid formation

Núria Benseny-Cases[1,3], Theodoros K. Karamanos[1,4], Cody L. Hoop[2], Jean Baum[2,*] & Sheena E. Radford[1,*]

From the [1]Astbury Centre for Structural Molecular Biology and School of Molecular and Cellular Biology, Faculty of Biological Sciences, University of Leeds, Leeds LS2 9JT, UK and [2]Department of Chemistry and Chemical Biology, Rutgers University, Piscataway, New Jersey, 08854, USA.

Figure S1

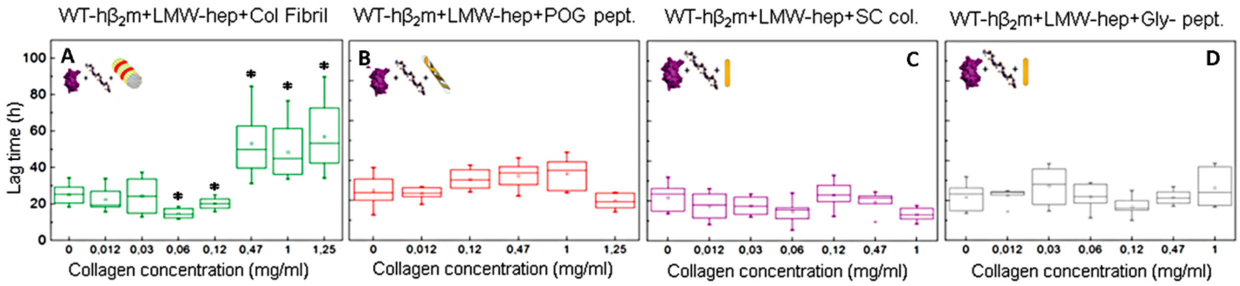

Figure S1. Fits of the lag time corresponding to the data shown in Figures 1 and 2. WT-hβ_2m (40μM) in the presence of 0.1 mg/ml LMW-heparin and different concentrations of (A) collagen I, (B) POG_{10}, (C) collagen I in single chain form, and (D) Gly-. Three replicate experiments, with three samples in each were measured. Asterisk denotes $p<0.02$.

Figure S2

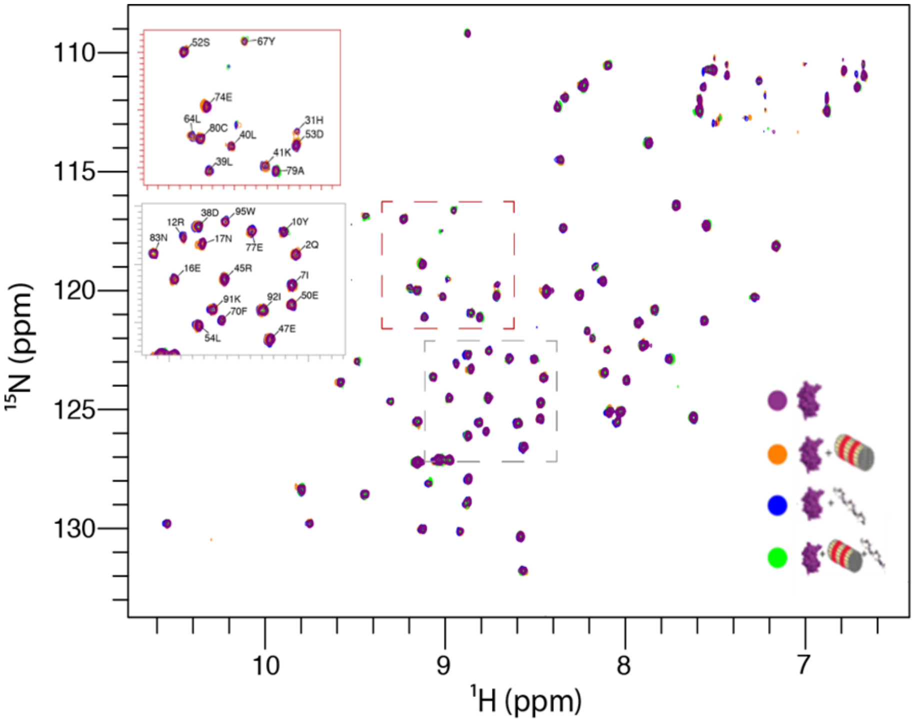

Figure S2. ^{1}H-^{15}N HSQC NMR spectra of WT-hβ_2m (80 μM) in the absence or presence of collagen I (2.0 mg/ml) and/or LMW-heparin (0.2 mg/ml). The insets are the zoomed-in areas inside the red and the blue boxes. Spectra of WT-hβ_2m (purple), WT-hβ_2m in the presence of collagen I (orange), WT-hβ_2m in the presence of LMW-heparin (blue) and WT-hβ_2m in the presence of collagen I and LMW-heparin (green).

Figure S3

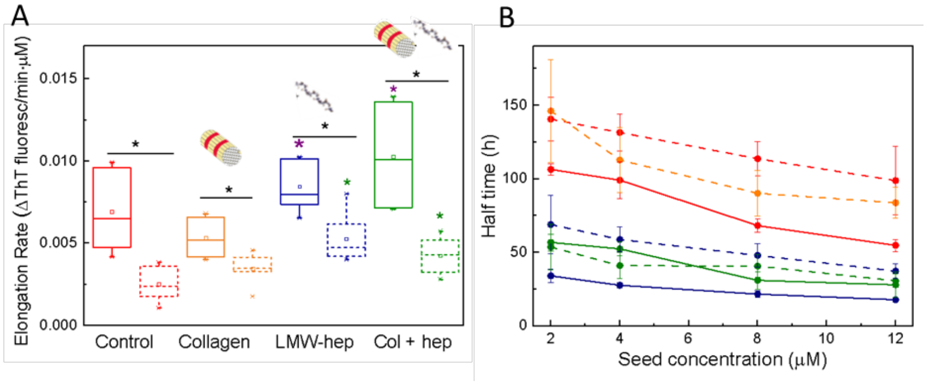

Figure S3. Kinetics of the elongation phase and the t_{50} of the entire reaction corresponding to the aggregation curves represented in Figures 4 and 5. (A) Elongation rate and (B) Half-time (t_{50}) of the progress curves of fibril formation using WT-hβ_2m seeds (continuous lines) or ΔN6-hβ_2m fibril seeds (discontinuous lines). In (A) and (B) the conditions used and data are labelled and colored as follows: WT-hβ_2m monomers (Control, red), WT-hβ_2m monomers in the presence of collagen I (Collagen, orange), in the presence of LMW-heparin (LMW-hep, blue) or in the presence of both LMW-heparin and collagen I (Col+hep, green). Purple asterisks show $p<0.05$ comparing elongation rates for self-seeded reactions relative to the rate in the absence of co-factors. Green asterisks show $p<0.05$ for cross-seeded reactions relative to the data in the absence of co-factors. Black asterisks show $p<0.05$ comparing the different conditions for the different type of seeds. Note that the t_{50} for WT-hβ_2m fibril growth seeded by WT-hβ_2m seeds in the presence of collagen I is not shown here since the progress curve did not reach the t_{50} in the time allotted. Three replicate experiments with three samples in each were acquired.
